# Comparative analysis of sampling and detection methods for fungal contamination on common healthcare environment surface materials

**DOI:** 10.1017/ice.2024.89

**Published:** 2024-10

**Authors:** Bobby G. Warren, Amanda Graves, Aaron Barrett, Alicia Nelson, Matthew Stiegel, Becky Smith, Ilan Schwartz, Deverick J. Anderson

**Affiliations:** 1 Duke Center for Antimicrobial Stewardship and Infection Prevention, Durham, NC, USA; 2 Disinfection, Resistance, and Transmission Epidemiology (DiRTE) Laboratory, Durham, NC, USA; 3 Division of Infectious Diseases, Duke University Medical Center, Durham, NC, USA; 4 Duke Occupational and Environmental Safety Office, Durham, NC, USA

## Abstract

We evaluated sampling and detection methods for fungal contamination on healthcare surface materials, comparing the efficacy of foam sponges, flocked swabs, and Replicate Organism Detection And Counting (RODAC) plates alongside culture-based quantification and quantitative polymerase chain reaction (qPCR). Findings indicate that sponge sampling and qPCR detection performed best, suggesting a foundation for future studies aiming to surveillance practices for fungi.

## Introduction

Healthcare-associated fungal infections and outbreaks are on the rise.^1,2^ At present, over 75,000 hospitalizations for fungal infections occur each year in the United States, primarily aspergillosis and candidiasis. The surveillance of healthcare environments for invasive fungal species is a critical component in infection control, particularly in preventing or responding to outbreaks. Current practices in environmental surface sampling, however, face significant limitations due to the absence of established threshold values and standardized methodologies.^3,4^ The objective of this study was to address these limitations by systematically comparing the efficacy of various sampling and detection techniques in a controlled experimental setting. By identifying the most effective methods, we sought to establish a foundation for standardized practices in environmental surveillance of fungal species within healthcare settings.

## Methods

We completed a comparative experimental design to evaluate different sampling and detection methods for fungal contamination on common healthcare surface materials, including aluminum, formica, linen, and high efficiency particulate air (HEPA) material, each measuring 10 × 10 cm. We uniformly inoculated approximately 10^4 colony-forming units (CFUs) of *Aspergillus fumigatus* (ATTC 204305) or *Candida parapsilosis* (ATCC 22019)) on 10 × 10 cm of each type of surface. Inocula were prepared using standard curves with OD600 and CFU/mL metrics. Standard spore collection was completed for *A. fumigatus* and liquid culture for *C. parapsilosis*, both using Sabouraud dextrose (SD) media. OD values were used to generate desired CFU/mL values, and serial dilutions were used to confirm CFU/mL. Following inoculation, the surfaces were allowed to air-dry for 10 minutes at ambient temperature to simulate real-world conditions. Each surface was evaluated 240 times (120 for *A. fumigatus* and 120 for *C. parapsilosis*).

Three primary sampling techniques were employed: 1) polyurethane sponge sticks, 2) nylon flocked swabs (both pre-moistened with a neutralizing buffer), and 3) Replicate Organism Detection And Counting (RODAC) plates containing SD agar. Sponges were positioned on the sampling surface ensuring full contact of one side of the sponge and then used to scrub the entire surface. Sponges were then flipped to ensure the opposite side made complete contact, and the entire surface was scrubbed again. Swabs were positioned similarly and scrubbed the entire surface while constantly rotating the swab to ensure usage of the entire swab surface. Replicate Organism Detection And Counting (RODAC) plates were directly placed onto the surface and carefully removed to avoid smearing. The recovery process for sponges involved the stomacher technique: sponges and 45 mL of phosphate-buffered saline with 0.1% Tween 20 were combined and stomached (Seward, Bohemia, NY, USA) at 260 RPM for 60 seconds. Resulting homogenates were centrifuged at 3100 rpm for 15 minutes, and all but 5 mL of the supernatant was decanted.^5^ Swabs were processed by vortexing in 1 mL of phosphate buffered saline (PBS). The eluents obtained from both sponges and swabs were then subjected to two detection methods: culture-based quantification and quantitative polymerase chain reaction (qPCR) using the FungiQuant primers for the fungal 18S rRNA gene in the QIAquant 96-5plex real-time PCR thermal cycler (Qiagen, Germany).^6,7^ Select PCR products from each experiment were sent for sequencing to rule out contamination. A standard curve was generated for both species between qPCR cycle thresholds and culture-based quantifications. Measured qPCR cycle thresholds were then used to calculate expected culture-based quantification to control for the increased sensitivity of qPCR compared to culture. The primary metric for comparison was the percent recovery of organisms, calculated as the total recovered sample area CFU relative to the known inoculum CFU. CFU were calculated from RODAC plates by counting plate CFUs, converting to CFU/cm^2^, and extrapolating that value to the size of the surface (100 cm^2^).

On each experiment day, all possible combinations of species, surface, sampling methodology, and detection methodology were run to control temporal variation. The Wilcoxon signed rank test was used to compare all CFU measurements, and *t* tests were used for qPCR cycle threshold comparisons. *P* < .05 was considered to be significant. All statistical tests were two-tailed and performed using SAS, version 9.4M7 (SAS Institute Inc).

## Results

In total, 960 samples were analyzed across multiple variables: 20 replicates with two fungal species, four study surfaces, three sampling methods, and two detection techniques.

Among sampling methods, foam sponges outperformed flocked swabs. The median percent recovery for sponges was 17.9% (IQR: 11.4–30.0) and 3.8% (IQR: 1.9–6.7) for swabs using culture-based detection (*P* < .01), and 36.2% (IQR: 25.7–78.4) and 10.5% (IQR: 7.7–36.0) using qPCR (*P* < .01), respectively. Replicate Organism Detection And Counting (RODAC) plates showed a median percent recovery of 3.4% (IQR: 1.0–7.1) via culture, suggesting limited efficacy compared to the other methods (Table 1).

**Table 1.** Fungal Recovery by Sampling Methodology and Detection Methodology

		RODAC		Swab					Sponge						
		Culture Median CFU (IQR)	Culture Recovery Proportion (IQR)	Culture Median CFU (IQR)	Culture Recovery Proportion (IQR)	qPCR CT (SD)	qPCR Recovery Proportion (IQR)	*p* Culture v qPCR	Culture Median CFU (IQR)	Culture Recovery Proportion (IQR)	qPCR CT (SD)	qPCR Recovery Proportion (IQR)	*p* Culture v qPCR	*p* Swab v Sponge Culture	*p* Swab v Sponge qPCR
*Both species*	Overall	552 (261–1007)	3.4 (0.8–7.1)	300 (200–700)	3.8 (1.9–6.7)	30.8 (1.1)	10.5 (7.7–36.0)	<0.01	2850 (900–4800)	17.9 (11.4–30.0)	29.5 (0.9)	36.2 (25.7–78.4)	<0.01	<0.01	<0.01
	Linen	144 (65–215)	2.8 (1.5–3.6)	200 (100–300)	3.3 (2.8–6.8)	32.0 (1.0)	13.7 (7.4–26.1)	<0.01	750 (525–975)	13.3 (10.0–20.5)	30.3 (0.9)	54.6 (25.7–117.4)	<0.01	<0.01	<0.01
	Formica	964 (864–1086)	10.6 (2.6–13.6)	1000 (500–1850)	5.8 (3.8–7.1)	31.0 (0.7)	9.8 (7.3–12.8)	<0.01	5850 (2883–9350)	29.8 (27.3–38.1)	29.0 (0.6)	40.2 (32.1–62.3)	<0.01	<0.01	<0.01
	Aluminum	914 (540–1149)	5.1 (3.4–9.4)	300 (200–475)	1.9 (0.6–6.3)	29.6 (0.8)	30.6 (7.9–130.5)	<0.01	1900 (725–4675)	13.3 (8.6–16.6)	29.1 (0.4)	47.8 (20.7–110.9)	<0.01	<0.01	<0.01
	HEPA	276 (197–332)	0.9 (0.6–5.5)	600 (200–1175)	3.1 (1.8–5.5)	30.6 (0.3)	19.2 (4.8–36.4)	<0.01	3800 (2925–4976)	20.2 (9.4–59.5)	29.6 (1.0)	32.3 (21.1–39.6)	0.58	<0.01	<0.01
*A. fumigatus*	Overall	406 (242–822)	7.0 (4.2–11.0)	300 (200–500)	5.0 (3.3–8.3)	31.1 (1.5)	24.5 (10.4–55.3)	<0.01	1,473 (700–3184)	23.5 (11.7–48.4)	30.1 (0.9)	44.8 (30.7–78.6)	<0.01	<0.01	<0.01
	Linen	214 (200–246)	3.6 (3.3–4.1)	200 (100–300)	3.3 (1.7–5.0)	32.8 (0.5)	7.5 (5.4–10.2)	<0.01	700 (525–800)	11.7 (8.8–13.3)	31.1 (0.2)	25.8 (21.9–27.7)	<0.01	<0.01	<0.01
	Formica	1,032 (941–1,119)	12.9 (11.8–14.0)	500 (225–675)	6.3 (2.8–8.4)	31.7 (0.3)	12.0 (10.6–14.1)	<0.01	2,968 (2,318–3,750)	37.1 (29.0–46.9)	29.5 (0.4)	61.6 (46.8–76.6)	<0.01	<0.01	<0.01
	Aluminum	552 (451–635)	9.2 (7.5–10.6)	350 (225–500)	5.8 (3.8–8.3)	29.0 (0.7)	121.0 (74.9–176.6)	<0.01	750 (500–900)	12.5 (8.3–15.0)	29.2 (0.4)	110.9 (79.4–134.5)	<0.01	<0.01	0.25
	HEPA	299 (238–350)	5.4 (4.3–6.4)	300 (100–600)	5.5 (1.8–10.9)	30.7 (0.2)	36.4 (33.2–42.3)	<0.01	3,250 (2,220–4,675)	59.1 (40.0–85.0)	30.6 (0.2)	39.5 (35.1–44.0)	<0.01	<0.01	0.22
*C. parapsilosis*	Overall	592 (94–1060)	2.4 (0.8–3.2)	500 (200–1475)	2.5 (1.4–4.8)	30.5 (0.5)	7.9 (6.3–14.5)	<0.01	4350 (1650–7325)	16.1 (9.9–26.8)	28.9 (0.5)	29.7 (21.1–70.4)	<0.01	<0.01	<0.01
	Linen	66 (52–83)	1.5 (1.2–1.9)	200 (100–300)	4.5 (2.3–6.8)	31.1 (0.6)	25.5 (20.2–43.4)	<0.01	900 (525–1175)	20.5 (11.9–26.7)	29.4 (0.3)	116.3 (90.8–137.4)	<0.01	<0.01	<0.01
	Formica	874 (840–966)	2.6 (2.5–2.9)	1800 (1425–2075)	5.5 (4.3–6.3)	30.3 (0.3)	7.3 (6.3–8.7)	<0.01	9300 (8750–9900)	28.2 (26.5–30.0)	28.5 (0.2)	33.4 (26.8–37.2)	0.02	<0.01	<0.01
	Aluminum	1142 (1094–1180)	3.5 (3.3–3.6)	200 (100–400)	0.6 (0.3–1.2)	30.2 (0.1)	7.9 (7.7–8.3)	<0.01	4650 (3800–5650)	14.1 (11.5–17.1)	29.0 (0.4)	20.9 (15.7–30.3)	<0.01	<0.01	<0.01
	HEPA	246 (189–319)	0.6 (0.4–0.7)	1100 (650–1550)	2.5 (1.5–3.5)	30.4 (0.4)	4.8 (4.0–6.9)	<0.01	4150 (3450–5175)	9.4 (7.8–11.8)	28.6 (0.2)	21.1 (17.9–25.6)	<0.01	<0.01	<0.01

Note: HEPA, high efficiency particulate air; RODAC, Replicate Organism Detection And Counting.

For detection methods, qPCR-based detection demonstrated a median percent recovery of 26.7% (IQR: 10.5–48.1), significantly higher than the 6.4% (IQR: 2.8–12.9) observed with culture-based methods (*P* < .01). This finding remained consistent within sponges as a sampling method with higher recovery from qPCR-based detection (36.2% [IQR: 25.7–78.42]) compared to culture (17.9 [IQR: 11.4–30.0]) (*P* < .01)) and within swabs (10.5% [IQR: 7.7–36.0]) compared to culture (3.8% [IQR:1.9–6.7]) (*P* < .01). When examining the recovery rates across different study surfaces, qPCR consistently showed higher recovery than culture-based methods. (Table 1).

## Discussion

Although surveillance of healthcare environments for invasive fungal species is critical to infection prevention, no current standards exist to guide surface sampling and detection methodologies for fungi whereas the field is more advanced for bacteria.^3,4,8,9^ The findings of this study have significant implications for environmental surveillance practices in healthcare settings. The superior performance of qPCR over culture-based methods indicates that qPCR should be considered a more reliable technique for detecting fungal contaminants. Our results are particularly relevant given the rapid and sensitive detection required in healthcare environments to prevent and control fungal outbreaks. The efficacy of sponge sampling in both culture and qPCR methods also highlights its potential as a preferred tool for environmental surveillance.

However, our study has limitations. The controlled experimental conditions may not fully replicate the complexities and variability found in real-world healthcare settings. Furthermore, the study’s focus on two fungal species, while relevant, does not encompass the full spectrum of potential fungal pathogens. Also, our experiments only used a single species on a surface, whereas in a real-world environment we would expect many species to be present and detected. Next, qPCR targeting the 18s gene is inferior to internal transcribed spacer (ITS) PCR for fungal species identification;^10^ however, we aimed to create a sampling and detection protocol that would capture all fungi in the sample. 18s can detect multiple fungi in the sample that can then be identified by next generation sequencing (NGS) and a microbiome analysis, whereas ITS requires some level or targeting or knowledge of what is in the sample to choose primers for the nested PCR as running all known nested ITS primers on individual samples would not be feasible. Lastly, quantitative analyses are currently not available utilizing qPCR.

In conclusion, our study presents a comprehensive analysis of various sampling and detection methods for fungal contamination on common materials used in healthcare environments. Our findings suggest that sponge sampling, combined with qPCR-based detection, leads to improved pathogen recovery compared to traditional culture-based methods. These insights lay the groundwork for future research aimed at establishing standardized environmental surveillance practices in healthcare settings, especially for invasive fungal species. Further studies are needed to validate these methods in real-world conditions and to develop practical threshold values for effective outbreak prevention and response strategies in healthcare environments.
